# New Pharmacological Approaches for Rare Diseases

**DOI:** 10.3390/ijms24087275

**Published:** 2023-04-14

**Authors:** Silvia Ortega-Gutiérrez

**Affiliations:** Departamento de Química Orgánica, Facultad de Ciencias Químicas, Universidad Complutense de Madrid, E-28040 Madrid, Spain; siortega@ucm.es

The expression ‘‘rare disease’’ describes a group of diseases whose individual prevalence is low (between 3.9 and 6.6 in 10,000 subjects depending on the country) but which in total affect up to the 3–6% of the worldwide population. The low prevalence of each disease represents an obstacle for the development of individually focused research programs, which are often limited by the scarcity of biological samples and the difficulty to access complete patient databases to perform statistically sounded preclinical and clinical studies. This fact translates into insufficient medical expertise and, consequently, into inadequate care offerings for these patients. Hence, the rare diseases field represents a currently unmet clinical need and provides, at the same time, an exciting area for basic and applied research. The intention of this Special Issue on “New Pharmacological Approaches for Rare Diseases” is to promote awareness of the field and to provide an up-to-date perspective of those emerging therapeutic strategies that are nowadays under active development and have the potential to generate, in the upcoming years, new pharmacological treatments to face these kind of largely ignored and usually fatal diseases. Around 80% of rare diseases are of genetic origin and, of those, 70% already start in childhood. Among them, muscular dystrophies (MDs) constitute an important class. The term encompasses more than 30 genetic diseases characterized by the progressive weakness and degeneration of the skeletal muscles that control movement [1]. In this field, Bencze provides an overview of the most important mechanisms of myofiber death in MDs [2]. New ongoing therapeutic approaches for treating specific MDs are also discussed for application in myotonic dystrophy type 1 (DM1), the most common form of adult MD, in Duchenne MD [3], or in limb-girdle MD R1 calpain 3-related (LGMDR1). These strategies explore different drug discovery approaches, including drug repurposing of the antitumor drug vorinostat for DM1 [4], the potential of natural products such as ectoine or of biologicals such as BLS-M22 for Duchenne MD [5,6], or the validation of glycogen synthase kinase 3β (GSK-3β) as a new therapeutic target for LGMDR1 [7]. Emerging therapies for other genetic rare diseases such as inherited peripheral neuropathies, collectively called Charcot–Marie–Tooth disease (CMT), are also covered [8]. Although CMT is considered rare, it is the most common hereditary neuropathy within neuromuscular diseases affecting approximately 1 in 2500 people.

Rare diseases affect all organs, including the immune system, and significant examples include autoimmune diseases such as neuromyelitis optica spectrum disorders (NMOSD), characterized by acute inflammation of the optic nerve and the spinal cord, and T-cell-associated tumors, such as acute lymphoblastic leukemia (ALL), which is the most common pediatric malignancy and of which T-cell ALL (T-ALL) comprises 10–15% of cases. In this Special Issue, Giglhuber and Berthele describe the main adverse events associated with the current treatments of NMOSD aiming at rationalizing treatment choices on an individualized basis by taking into account the safety concerns associated with the different drugs [9], and Lato et al. review the recent development of targeted therapeutic approaches for T-ALL, illustrating the growing potential of personalized medicine based on thorough molecular profiling [10]. Additionally, drug repurposing could represent another strategy with potential for identifying treatments for underexplored diseases such as nephropathic cystinosis, a lysosomal storage disease that eventually can lead to renal failure [11]. Some rare diseases, such as retinopathy of prematurity (ROP), an ocular disorder in preterm infants, have increased gradually [12], whereas others, as is the case for cystic fibrosis, have reduced their incidence [13] due to advances in the field [14]. Finally, among very rare diseases, the Hutchinson–Gilford progeria syndrome (HGPS) or progeria, a pathology that affects all organs except the central nervous system characterized by a global accelerated aging phenotype, stands out [15]. Progeria exemplifies how scientific research can significantly improve the outcome of a disease, from the characterization of the molecular cause and the precise description of associated cellular defects to the first marketed drug for its treatment in two decades [16,17,18,19], with important biomedical advances related with gene therapy awaiting on the near horizon [20].

Among the sporadic rare diseases, that is, with random occurrence and with no clear associated risk factors nor family history of the disease, amyotrophic lateral sclerosis (ALS) deserves special attention. ALS affects motor neurons that control voluntary muscle movement and is considered a fatal disease since it lacks a cure or even an effective treatment to reverse or effectively delay its progression [21]. In this context, the relevance of mitophagy modulation [22] and other molecular alterations [23] together with the specific role of chaperone protein BiP [24] in ALS have been explored. These findings could open uncharted directions that may aid in the identification of novel therapeutic strategies for facing this devastating disorder.

Rare diseases represent a very complex field that, for its very own features, entails enormous economic and societal implications. Nonetheless, as has been demonstrated through recent history, research provides continuous advances. We are confident that the work covered in this Special Issue contributes to stimulate research on this fascinating area that can translate, in the near future, into new therapies, meaning we will be able to provide hope to the millions of patients currently suffering from a rare disease.
